# The role of autopsy on the diagnosis of missed injuries and on the trauma quality program goal definitions: study of 192 cases

**DOI:** 10.1590/0100-6991e-20223319_en

**Published:** 2022-11-10

**Authors:** AUGUSTO CANTON GONÇALVES, JOSÉ GUSTAVO PARREIRA, VICTOR ALEXANDRE PERCINIO GIANVECCHIO, PEDRO DE SOUZA LUCARELLI-ANTUNES, LUCA GIOVANNI ANTONIO PIVETTA, JACQUELINE ARANTES GIANNINNI PERLINGEIRO, JOSE CESAR ASSEF

**Affiliations:** 1 - Faculdade de Ciências Médicas da Santa Casa de São Paulo, Departamento de Cirurgia - São Paulo - SP - Brasil; 2 - Irmandade da Santa Casa de Misericórdia de São Paulo, Serviço de Emergência - São Paulo - SP - Brasil; 3 - Instituto Médico Legal de São Paulo - São Paulo - SP - Brasil

**Keywords:** Autopsy, Trauma Severity Indices, Missed Diagnosis, Multiple Trauma, Autopsia, Índices de Gravidade do Trauma, Diagnóstico Ausente, Traumatismo Múltiplo

## Abstract

**Objective:**

to assess the role of autopsy in the diagnosis of missed injuries (MI) and definition of trauma quality program goals.

**Method:**

Retrospective analysis of autopsy reports and patient’s charts. Injuries present in the autopsy, but not in the chart, were defined as “missed”. MI were characterized using Goldman’s criteria: Class I, if the diagnosis would have modified the management and outcome; Class II, if it would have modified the management, but not the outcome; Class III, if it would not have modified neither the management nor the outcome. We used Mann-Whitney’s U and Pearson’s chi square for statistical analysis, considering p<0.05 as significant.

**Results:**

We included 192 patients, with mean age of 56.8 years. Blunt trauma accounted for 181 cases, and 28.6% were due to falls from the same level. MI were diagnosed in 39 patients (20.3%). Using Goldman’s criteria, MI were categorized as Class I in 3 (1.6%) and Class II in 11 (5.6%). MI were more often diagnosed in the thoracic segment (25 patients, 64.1% of the MI). The variables significantly associated (p<0.05) to MI were: time of hospitalization < 48 h, severe trauma mechanism, and not undergoing surgery or computed tomography. At autopsy, the values of ISS and NISS were higher in patients with MI.

**Conclusion:**

the review of the autopsy report allowed diagnosis of MIs, which did not influence outcome in their majority. Many opportunities of improvement in quality of care were identified.

## RESUMO

**Objetivo:**

Avaliar a utilidade da autópsia no diagnóstico de lesões despercebidas (LD) e no estabelecimento de metas para programa de qualidade em trauma.

**Método:**

análise retrospectiva dos laudos de autópsia por trauma entre outubro/2017 e março/2019 provenientes do mesmo hospital. Lesões descritas na autópsia, mas não no prontuário médico, foram consideradas como despercebidas (LD) e classificadas pelos critérios de Goldman: Classe I: mudariam a conduta e alterariam o desfecho; Classe II: mudariam a conduta, mas não o desfecho; Classe III: não mudariam nem a conduta nem o desfecho. As variáveis coletadas foram comparadas entre o grupo com LD e os demais, através de método estatístico orientado por profissional na área. Consideramos p<0,05 como significativo.

**Resultados:**

analisamos 192 casos, com média etária de 56,8 anos. O trauma fechado foi o mecanismo em 181 casos, sendo 28,6% por quedas da própria altura. LD foram observadas em 39 casos (20,3%), sendo 3 (1,6%) classe I e 11 (5,6%) classe II. O tórax foi o segmento com maior número de LD (25 casos - 64,1% das LD). Foram associados à presença de LD (p<0,05): tempo de internação menor que 48 horas, mecanismo de trauma grave e a não realização de procedimento cirúrgico ou tomografia. Nos óbitos até 48h, valores de ISS e NISS nas autópsias foram maiores que os da internação.

**Conclusão:**

a revisão das autópsias permitiu identificação de LD, na sua maioria sem influência sobre conduta e prognóstico. Mesmo assim, várias oportunidades foram criadas para o programa de qualidade.

## INTRODUCTION

Trauma has been recognized for decades as one of the main causes of death and socioeconomic impact worldwide^1^. According to DATASUS, in 2019 trauma was responsible for 142,800 deaths in Brazil^2^. The most frequent causes are traumatic brain injury, followed by hemorrhage and associated injuries, resulting from traffic accidents and interpersonal violence^3^ ^,^ ^4^. 

Trauma is a “perfect storm”, capable of inducing the most experienced physician to error^5^. There are factors that predispose to failure, such as handling unstable patients without the necessary information, being forced to make decisions promptly and in a limited time, as well as dealing with multiple tasks and different teams simultaneously^5^. The result is reported in several studies, which describe adverse events in detail, even in the best trauma centers and mature care systems^5^ ^-^ ^8^. Thus, the implementation of quality programs is essential^9^.

The review of deaths and failures in care is an important tool in quality programs. Vioque et al., in 2014, reviewed 377 deaths in trauma victims, classifying 106 cases (28%) as “preventable” or “potentially preventable”^7^. Teixeira et al., in 2007, classified the failures as resulting from treatment delay, clinical judgment errors, technical problems, and missed injuries (MI)^8^.

The analysis of unnoticed injuries is important to understand what really happened to the patient^10^ ^,^ ^11^. The autopsy proved to be an important tool in the identification of undiagnosed lesions, which occur in 10% to 47% of hospital deaths in trauma victims^11^ ^-^ ^18^. The Brazilian penal code requires autopsy of all cases of suspected or unnatural death^19^. Despite the law promoting a high number of autopsies, we did not find many national studies comparing the ante-mortem and post-mortem findings of trauma victims^19^. 

Our study aims to analyze the value of autopsy in identifying unnoticed lesions and their characteristics, as well as in its use in defining goals for a quality program

## METHODS

This study was approved by the Ethics in Research Committee of our hospital under registration CAAE: 24878919.0.0000.5479.

We carried out a retrospective analysis of trauma autopsy reports performed at the Instituto Médico Legal - SP (São Paulo state Coroner’s Office - IML), of cases treated between October 2017 and March 2019 from the same hospital. In each autopsy, we observed the descriptive report, the reported injuries, and the cause of death. Each case had its hospital chart reviewed, with the aim of comparing information on admission with the autopsy report. We excluded patients admitted in cardiorespiratory arrest, cases of readmission due to post-trauma complications, those with insufficient data, and those with diagnoses of unconfirmed trauma (due to the absence of traumatic injury at admission and at autopsy).

We collected data on demographics, trauma mechanism, initial management, identified injuries and their treatment, and time between admission and death. All injuries observed during hospitalization, as well as those described in the autopsy reports, were stratified according to the Abbreviated Injury Scale 2015 (AIS 2015), the Injury Severity Score (ISS), and the New Injury Severity Score (NISS)20-22. Briefly, the AIS scale classifies injuries into 6 degrees, with AIS≥3 being deemed severe.

We considered the variable “length of stay” (LS) as the period between admission to our hospital and death. The variable “time between first care and death” (TBFCD) refers to the time between the first care in the hospital of origin until death in our hospital, being calculated in patients transferred from another service.

A pair of reviewers (surgeons) analyzed the medical records of eligible patients and their autopsy reports to define the presence of unnoticed lesions and their impact on outcome. In case of disagreement, a third reviewer was called upon to give his opinion and settle the issue. An injury was considered “missed” when, based on its observation in the autopsy report, it could not be identified in the medical record.

The impact of missed lesions on the outcome was estimated from the modification of Goldman’s clinicopathological criteria, a method also used by Ong et al. and Light et al.^14^ ^,^ ^23^ ^,^ ^24^.

- Class I: injuries that, if diagnosed, would possibly change the conduct and alter the outcome;
- Class II: injuries that, if diagnosed, would possibly change the conduct, but would not change the outcome;
- Class III: injuries that would change neither the conduct nor the outcome; and
- Class IV (this item is an addition by the authors of this study, due to the impossibility of classification based on available information): patient has a missed injury, but there are no data to classify it.

If more than one MI was identified in a patient, the Goldman classification would be noted for the most severe lesion.

We performed a comparison of the variables collected between two groups:

a) Group with MI: patients with unnoticed injuries characterized according to the above criteria, except for those with class III injuries and AIS=1, as these corresponded to minimal injuries, without clinical significance, which were possibly not valued in the context of severe trauma (eg. right thigh bruise); and
b) Group without MI: other patients.

Patients with “unclassifiable” lesions at autopsy, either due to lack of data or to treatment at admission (eg, patient with splenic lesion treated by splenectomy) were excluded from the ISS and NISS calculation.

Statistical analysis was conducted by a biostatistician, together with the authors. Data were presented as means, standard deviations, and minimum and maximum values of scores for quantitative variables, and proportions for qualitative ones. We performed the Shapiro-Wilk test to verify the adherence of quantitative variables to the normal distribution, determining the types of statistical tests to be used. For categorical variables, we performed association analyzes using the Pearson’s chi-square test. If there was any variable with an expected frequency lower than five, we used the chi-square test with Yates’ correction. To compare the means of quantitative, dichotomous variables, we used the Mann-Whitney U test. In all analyses, we adopted the descriptive level of p<0.05. For the purposes of univariate analyses, we grouped some qualitative variables in categories and categorized some quantitative ones, using the frequency distributions and/or observed risk and/or literature cutoffs as criteria. To perform the statistical analysis, we used the STATA software, version 14.

## RESULTS

We initially included 340 patients. We excluded 31 cases referred to the IML as trauma victims but without traumatic injuries identified at autopsy, 11 because they were readmissions, 19 due to insufficient data, and 87 patients who arrived at the hospital in cardiorespiratory arrest.

**Figure 1:**
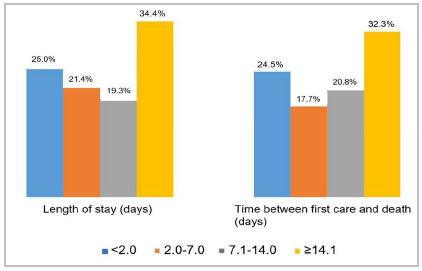
Length of stay and time between first care and death.

The sample consisted of 192 cases, 78.1% male, with a mean age of 56.8 years. Eighty-five patients (44.3%) were 60 years of age or older (Table 1). The most frequent trauma mechanism was fall from the same level, in 55 cases (28.6%) (Table 1). Thirty-eight patients (19.8%) were transferred from other hospitals. The time between hospitalization and death was shorter than two days in 48 (25.0%) cases and longer than 14 days in 66 (34.4%) (Figure 1). Computed tomography was performed in 155 (81.0%) patients and 90 (47.0%) underwent some surgical procedure.

**Table 1:** Distribution of 192 patients according to age group and trauma mechanism.

Feature	nº	%
Age group		
under 30	16	8.3
30 to 39	24	12.5
40 to 49	33	17.2
50 to 59	34	17.7
60 to 69	34	17.7
70 to 79	20	10.4
80 and over	31	16.1
Main trauma mechanism		
Fall from the same level	55	28.6
Fall	33	17.2
Assault	12	6.3
Trampling	30	15.6
Car accident	2	1.0
Motorcycle accident	12	6.3
Bicycle accident	4	2.1
Stabbing wound	5	2.6
Gunshot wound	6	3.1
Fall from stairs	17	8.9
Unknown	10	5.2
Other	6	3.1

According to the medical records, 62.4% of the patients had lesions in the cephalic segment, 45.3% in the extremities, 20.9% in the chest, and 11.4% in the abdomen. Lesions with AIS≥3 were identified in the cephalic segment in 57.9%, in the extremities in 20.9%, in the chest in 13.0%, and in the abdomen in 6.8% (Figure 2A). Spinal cord trauma was identified in 14.2%. In the autopsies, lesions in the cephalic segment were identified in 65.7%, 54.7% with AIS≥3 (Figure 2B). Considering all group, the mean and standard deviation of the ISS calculated during hospitalization and at autopsy were 16.9±8.5 and 14.6±9.3, respectively. The NISS calculated during hospitalization was 24.5±14.0, and at autopsy, 21.0±12.3.

**Figura 2a:**
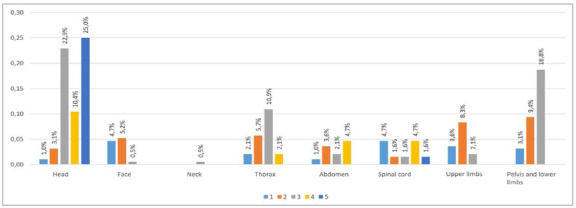
Injuries identified at admission, separated by body segment and Abbreviated Injury Scale (AIS).

**Figura 2b:**
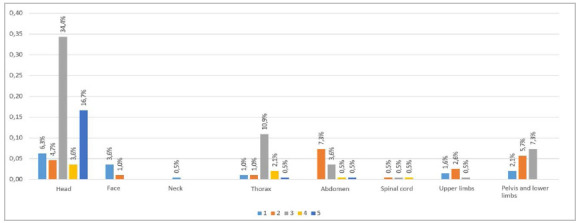
Lesions identified at autopsy, separated by body segment and Abbreviated Injury Scale (AIS).

Missed lesions were identified in 39 patients (20.3%). According to the Goldman criteria, 24 (12.5%) were class III, 11 (5.7%) were class II, and three (1.6%) were class I. One patient did not have sufficient data for classification (class IV). MI were most frequently identified in the chest (25 cases - 64.1%), head (13 cases - 33.3%), and abdomen (11 cases - 28.2%). When considering only Goldman I and II MI, the thorax was the most affected segment, with nine cases, followed by the skull (two), abdomen (two), and extremities (one) (Table 2).

**Table 2:** Distribution of missed lesions classified as Goldman I or II (summed) by anatomical segment, in the sample of 192 patients.

Segment	n	%
Head	2	1.0
Chest	9	4.7
Abdomen	2	1.0
Extremities	1	0.5

MI were significantly less frequent in victims of falls from the same level (9.1% vs. 24.8%, p=0.011). Falls from heights and running over displayed a significantly higher frequency of MI, 33.3% and 26.7%, respectively (Table 3). MI were less frequent in patients who underwent a surgical procedure (12.9% vs. 27.3%, p=0.013). The same occurred in the group that underwent computed tomography compared with the others (14.8% vs. 43.2%, p<0.001). MI occurred more frequently in the group of patients who died within 48 hours (47.9% vs. 11.1%, p<0.001) (Table 3).

**Table 3:** Analysis of personal characteristics and hospitalization, according to the presence of unnoticed injury.

Feature	No injury		With injury		Total		p*
	no.	%	no.	%	no.	%	
Length of stay (days)							
Less than 2.0	25	52.1	23	47.9	48	100.0	<0.001
2.0 to 7.0	36	87.8	5	12.2	41	100.0	
7.1 to 14.0	31	83.8	6	16.2	37	100.0	
14.1 and more	61	92.4	5	7.6	66	100.0	
Time between first care and death (in days)**							
Less than 2.0	24	51.1	23	48.9	47	100.0	<0.001
2.0 to 7.0	31	91.2	3	8.8	34	100.0	
7.1 to 14.0	33	82.5	7	17.5	40	100.0	
14.1 and more	57	91.9	5	8.1	62	100.0	
Trauma mechanism							
Fall from standing height	50	90.9	5	9.1	55	100.0	0.011
Fall	22	66.7	11	33.3	33	100.0	
Trampling	22	73.3	8	26.7	30	100.0	
Motorcycle and bicycle accidents, Stabbing and Gunshot wounds	18	66.7	9	33.3	27	100.0	
Other	41	87.2	6	12.8	47	100.0	
Surgical procedure							
No	72	72.7	27	27.3	99	100.0	0.013
Yes	81	87.1	12	12.9	93	100.0	
Computed tomography							
No	21	56.8	16	43.2	37	100.0	<0.001
Yes	132	85.2	23	14.8	155	100.0	
Total	153	79.7	39	20.3	192	100.0	

*Chi-square test; **Excluded 9 cases without information; p<0.05.

At autopsy, the values of ISS (20.9±10.9 vs. 12.4±7.6, p<0.001) and NISS (26.8±12.0 vs. 19.0±11.8, p<0.001) were higher in patients with MI. When analyzing deaths within 48 days after admission, the mean ISS (23.6±10.4 vs. 13.4±.2, p<0.001) at autopsy was also higher in patients with unnoticed injuries.

## DISCUSSION

We identified some important features in our analysis. Deaths were evenly distributed between age groups, with 44.3% in elderly patients. The mechanism falling from the same level was the most frequent, which can be explained by the presence of elderly patients^25^. Head injuries were the most frequent and the most severe, being the main cause of death. Dutton et al., in 2010, reported that 51.6% of deaths from trauma occurred due to intracranial injuries^26^. Our sample was also characterized by a low percentage of penetrating injuries compared with other Brazilian series^4^ ^,^ ^27^.

Trunkey, in 1983, described the trimodal distribution of deaths in trauma patients^28^. In the model, about 50% of deaths would occur immediately after trauma, 30% a few hours later, and the rest, later. The deaths of the second peak, understood as “early”, would be considered avoidable or potentially avoidable, directing efforts to improve the quality of care. In recent years, with the improvement of pre-hospital and hospital care, a change in this scenario has been observed, with a bimodal distribution or even a single peak of deaths, which would occur early after trauma^29^.

We observed that 25% of our cases died within 48 hours of hospitalization, which is precisely the most severe group, with the highest chance of having unnoticed injuries. However, more than half of the patients died after seven days of hospitalization. It is important to differentiate our study from those that analyze trauma deaths in general. Pre-hospital deaths, as well as patients in cardiac arrest at admission, were not part of our sample, which may explain the different results.

This fact can be understood by the study by Evans et al., who identified a different distribution of deaths according to trauma severity30. They observed a higher frequency of deaths within 48 hours for high-energy trauma (79.4%). For less complex mechanisms (falls of less than one meter), the relationship is reversed, with only 20% of deaths within 48 hours and 49% in more than seven days. Our data corroborates this trend, since most of our cases were of mechanisms with lower energy (falls from the same level), with low values of ISS and NISS.

The incidence of MI was comparable to other studies^11^ ^,^ ^12^ ^,^ ^15^ ^,^ ^18^ ^,^ ^31^. We observed MI in about 20.3% of our cases, with 35.9% being classes I and II. Albreksten and Thomsen described MI in 34.0% of 218 reviewed autopsies, 81.3% of which being deemed associated with the clinical outcome12. Sharma et al. identified 11.2% of MI in 842 autopsies, in a service with a high percentage of burns (25%)11. Steinwall et al. studied 132 deaths, observing an incidence of 10.6% of MI, 28.6% of which related to death^16^. Ong et al. found that 19% of post-trauma autopsy cases had some clinically relevant diagnosis not identified during hospitalization^14^. These findings reinforce the importance of performing an autopsy in trauma patients, as well as the analysis of their results in programs that aim to improve care.

Missed injuries occurred more frequently in the thoracic segment (64.1%), corresponding to 23.1% of class I and II lesions. Boudreau et al. and Steinwall et al. also observed more than a third of the missed lesions in the thoracic segment^16^ ^,^ ^18^, as in our study.

The AIS scale and the ISS and NISS severity scores are used to stratify the anatomic severity of injuries. Boudreau et al. described a mean increase of 38.9% in the ISS values at autopsy in relation to those recorded during hospitalization18. As in our study, we noted this difference in early deaths, but not in prolonged hospitalizations. These data also reinforce the importance of post-mortem analysis for understanding the patient’s evolution.

Our study has some limitations. Because it is retrospective, the medical records and autopsy data are not complete in many cases. The autopsy descriptions are often not compatible with the AIS, which required interpretation by the reviewers. The classification of lesions according to the AIS scale and stratification by classes is dependent both on the detail and clarity of the data source and on the ability of the reviewers to interpret them, which may cause bias. It is noteworthy that some AIS codes are based on clinical information, which hamper their use, especially in the analysis of autopsy data, in which descriptions are sometimes limited.

Perhaps the strongest point of our study is the comparison of the clinical course with the autopsy findings, which is not frequent in our country. Most studies aim to describe the causes of death and do not make a connection with hospital care. In previous work, we had the opportunity to study outcomes based on trauma indices, classifying deaths as preventable or not. With the result of the autopsy, there is a broader view of the problem and the issues to be addressed in the quality program.

A trauma quality program aims at the identification of opportunities to improve care, the planning and implementation of measures with this objective, and the reassessment to ensure the effectiveness of these initiatives. With the analysis of the data from this study, we can propose some actions to improve quality of care. Due to the presence of unnoticed lesions in 20.3% of deaths, we could propose a tertiary assessment (complete reassessment after 24h of admission), as a measure to reduce these numbers. As unnoticed injuries were more frequent in patients who did not undergo CT, protocols for performing this exam (eventually in unstable patients) need to be reviewed, to offer this opportunity to more severely ill patients. We also observed that MI occurred more frequently in trauma patients who were not operated on and in those with a shorter time between admission and death, reinforcing the idea that surgical decision-making is an important point for identifying these injuries. With this information, there is a need to train the team to quickly make decisions as an indication for tomography and resuscitation in the operating room. Finally, a striking fact in this study was the frequency of deaths in the elderly and in those with falls from the same level. This part of the sample had a longer hospital stay, with deaths often not directly related to traumatic injuries. The implementation of a specific care group to attend to these cases, with a multidisciplinary view and with the support of geriatricians, could be an option to reduce these numbers. Obviously, we cannot assume that these actions will necessarily have the expected result, or even that these measures are possible to be implemented. These are proposals that should be discussed with managers and care teams, certainly being submitted for evaluation of their results in future analyses.

The main message of this study is that the analysis of the autopsy, together with the evolution recorded in the medical chart, allowed the identification of unnoticed lesions, their classification, and suggestion of points to be worked on in a quality program.
